# Synergistically Reinforced Copper-Free Friction Materials with Agricultural Wastes and Carbon Fibers: Evaluation of Tribological Performance

**DOI:** 10.3390/ma19101941

**Published:** 2026-05-09

**Authors:** Yitong Tian, Kunsen Huang, Zihe Xu, Yuqi Zhuansun, Yunhai Ma

**Affiliations:** 1 The College of Biological and Agricultural Engineering, Jilin University, 5988 Renmin Street, Changchun 130025, China; tianytmails@163.com (Y.T.); huangks24@mails.jlu.edu.cn (K.H.); zhxu21@mails.jlu.edu.cn (Z.X.); zsyq23@mails.jlu.edu.cn (Y.Z.); 2 The Key Laboratory of Bionic Engineering, Ministry of Education, Jilin University, 5988 Renmin Street, Changchun 130025, China; 3 Institute of Structured and Architected Materials, Liaoning Academy of Materials, Shenyang 110167, China

**Keywords:** agricultural wastes, carbon fibers, tribological performance, copper-free friction materials

## Abstract

Driven by global environmental regulations that strictly limit copper content in brake pads, traditional copper-based friction materials face significant challenges due to their negative ecological impacts. Consequently, the development of sustainable, copper-free alternatives has become an inevitable trend in the braking industry. This study proposes a novel approach to developing high-performance green friction materials by utilizing a synergistic combination of agricultural wastes, specifically corn cobs, wheat straw, rice husks, and sugarcane bagasse, and carbon fibers. Research indicates that the friction coefficient of the synergistic formulation remains stable within the range of 0.35 to 0.48. Compared with the control group, this formulation achieves an average reduction in the wear rate of 19.28% and an increase in the recovery rate of 5.15%, demonstrating superior tribological performance. The synergistic interfacial regulation between carbon fibers and agricultural waste facilitates the construction of a smooth and stable friction layer, which maintains consistent performance during extended operating conditions. Among all formulations investigated, the composite reinforced by the synergy of corncob and carbon fiber exhibits the most prominent comprehensive properties, with the wear rate decreasing by 28.73% and the recovery performance improving by 4.05% relative to the specimen containing copper fibers. This work not only provides a new pathway for the sustainable development of green friction materials but also offers a theoretical basis for the high-value utilization of agricultural waste resources.

## 1. Introduction

Brake friction materials are core components that ensure transportation safety, and their tribological properties directly determine a vehicle’s braking performance and driving stability. In traditional formulations, copper has long been regarded as an indispensable key component due to its excellent thermal conductivity, thermal stability, and smooth friction characteristics [1,2]. However, copper-containing wear particles generated during frequent braking can enter water bodies and soil via rainwater, leading to the accumulation of hard-to-degrade heavy metals and causing lasting damage to the ecological environment [3,4,5]. With global environmental regulations imposing strict limits on copper content in brake pads, “copper-free” and “green” formulations have become an inevitable trend in the development of friction materials [6,7].

Recent studies have highlighted various strategies to replace copper in friction materials [8,9]. Metal particles have been investigated as potential substitutes in several works [10]. Similarly, synthetic fibers have also been proposed for this purpose [11,12,13,14]. In addition, many researchers have focused on solid lubricants such as graphite [15,16]. However, a single replacement material often struggles to meet multiple tribological requirements. For example, high-hardness metal particles can easily cause excessive cutting of the mating surface, while traditional polymer fibers are highly prone to thermal degradation under high-temperature conditions. Therefore, how to achieve copper-free systems while maintaining a stable coefficient of friction and extremely low wear rates remains a major challenge facing current tribological research.

To overcome these limitations, agricultural waste has emerged as a promising green reinforcing material due to its abundance, renewability, low density, and naturally porous or fibrous structure [17]. Previous studies have explored the use of agricultural wastes in friction materials, including crop straw, shells, and fruit pomace [18,19,20,21]. Incorporating these materials offers two main benefits: reducing production costs and minimizing air pollution caused by incineration. Olabisi et al. [22] demonstrated the technical feasibility of utilizing various agricultural residues—specifically palm kernel shells, maize husks, cocoa bean shells, and melon shells—as replacements for conventional components in friction materials. Their comparative analysis revealed that composites reinforced with maize husks exhibited superior overall performance, particularly achieving enhanced thermal stability, optimized porosity, and reduced wear rates. Consequently, these findings confirm the potential of agricultural wastes for the development of environmentally sustainable braking solutions. However, due to the relatively poor intrinsic thermal stability of biomass fibers, structural failure is prone to occur under high-load continuous braking conditions. This issue has drawn significant research attention [17,23,24]. One proposed approach is to blend various agricultural wastes. Oladele et al. [25] systematically evaluated the tribological performance of hybrid composites reinforced with pawpaw stem and glass fibers. Their findings demonstrated that the integration of vegetable fibers not only improved thermal conductivity and accelerated the curing process of the resin matrix but also significantly enhanced wear resistance. Notably, network-structured pawpaw fibers exhibited the most effective reinforcement at a loading of 15 wt%, clearly demonstrating the functional benefits derived from specific morphologies of biowaste. Carbon fiber is widely recognized as the ideal matrix for high-performance friction materials due to its outstanding specific strength, high modulus, and excellent thermal stability. Nevertheless, its inherent brittleness and high cost remain challenges, as excessive carbon fiber content can induce abrasive wear on the friction surface. For this reason, researchers often blend it with other types of fibers to balance performance and cost through synergistic effects. Common examples include glass fibers [26,27] and aramid fibers [28,29]. Related studies have confirmed the improvement in friction and wear properties [30]. Although there have been numerous reports on the blending of carbon fibers with synthetic fibers, systematic research on the synergistic reinforcement effects between carbon fibers and agricultural waste fibers remains relatively scarce. In particular, it is still unclear how the natural characteristics of agricultural waste interact with high-modulus carbon fibers at the friction interface and how this relates to the mechanisms of friction and wear.

Therefore, this study proposes a green, copper-free friction material system synergistically reinforced with agricultural waste and carbon fibers. Through a systematic investigation of how various types of agricultural waste, in combination with carbon fibers, influence the physicochemical and tribological properties of the material, this research provides an in-depth exploration of the underlying synergistic regulation mechanisms. This work not only opens up new avenues for the high-value utilization of agricultural waste in high-performance friction materials but also establishes a novel scientific basis and technical support for the formulation design of environmentally friendly, copper-free brake materials.

## 2. Materials and Methods

### 2.1. Materials and Fabrication

**Figure 1:**
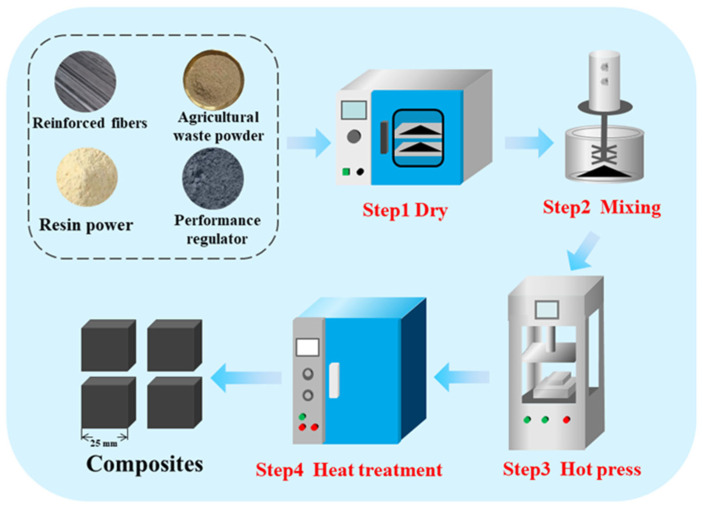
Schematic of the manufacturing process of braking friction materials.

The friction material prepared in this study consists of carbon fibers, agricultural waste powder, mineral fibers, flake graphite, phenolic resin, nitrile rubber powder, aluminum oxide, barium sulfate, petroleum coke and friction powder. The agricultural waste powder was sourced from an agricultural product processing plant in Changchun. The agricultural waste powders used in this study include corn cob powder, wheat straw powder, rice husk powder and sugarcane bagasse powder. Table 1 lists the components, and relevant information used in the development of the friction material. Figure 1 illustrates the preparation process for the brake friction materials. Initially, all components are placed in a JF850R (Wanda Machinery, Changchun, China) drying oven and dried for 90 min at 80 °C, after which they are thoroughly mixed using a JF810 (Wanda Machinery, Changchun, China) plow-type mixer in accordance with the mass fractions specified in Table 1. The resulting mixture is poured into a mold specifically designed for the production of brake friction materials and hot-pressed for 30 min at 160 °C and 40 MPa using a JFY50 (Wanda Machinery, Changchun, China) hot-press.

**Figure 2:**
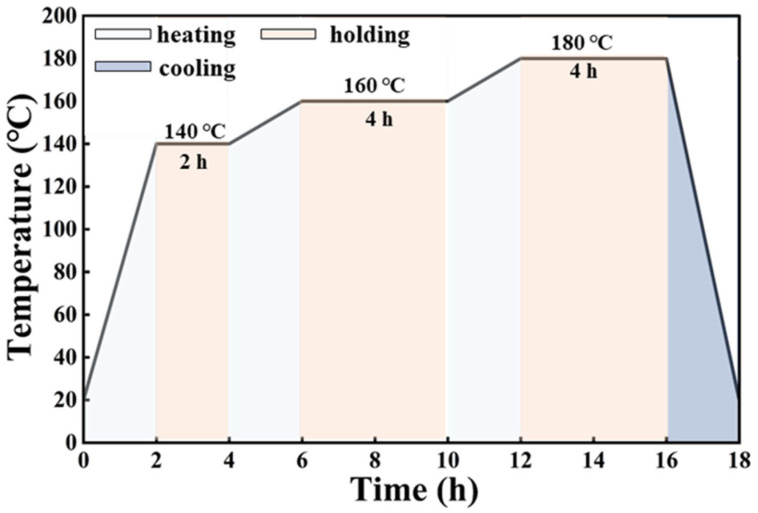
Heat treatment schematic.

The preformed friction material specimens undergo heat treatment to eliminate internal thermal stresses and ensure complete curing [31]. The heat treatment process is illustrated in Figure 2.

After hot pressing, the specimens are placed in an electric heating oven (JF980A Wanda Machinery, Changchun, China) for heat treatment. The specimens are cured at 140 °C for 2 h, followed by successive curing stages at 160 °C for 4 h and 180 °C for 4 h, before being allowed to cool naturally to room temperature. The heat-treated specimens are subsequently sectioned into friction and wear test specimens with dimensions of 25 mm × 25 mm × 6.5 mm.

The formulation content of the specimens is presented in Table 2. FM-0, FM-1, FM-2, FM-3 and FM-4 correspond to friction materials containing copper fibers, corn cob powder, wheat straw powder, rice husk powder and sugarcane bagasse powder, respectively. FM-0 serves as the control group. The letters ‘F’ and ‘M’ in the friction material specimens stand for ‘friction’ and ‘material’, respectively.

### 2.2. Physical and Mechanical Property Characterization

The density of the test specimens was determined using Archimedes’ principle of buoyancy. The hardness of the test specimens was measured using a Rockwell hardness tester (HRSS-150, Hangzhou, China). The impact strength was tested using an impact testing machine (XJ-40A, Jinan, China). The impact strength was tested in accordance with GB/T 33835-2017 [32], with test specimens cut to dimensions of 55 mm × 6 mm × 4 mm. The maximum impact energy, swing angle and impact velocity of the impact pendulum were 0.981 J, 150° and 2.9 m/s, respectively. Shear strength was tested using a universal testing machine (WAW-100, Guilin, China). Shear strength was tested in accordance with GB/T 22309-2023 [33], with test specimens cut to dimensions of 20 mm × 20 mm × 10 mm. Each specimen is tested five times, and the average value is taken as the test result.

### 2.3. Tribological Characterization

The friction and wear properties of FM-0, FM-1, FM-2, FM-3, and FM-4 were evaluated using a JF150D-II constant-speed friction and wear tester (Wanda Machinery, Changchun, China) in accordance with the Chinese National Standard GB 5763-2018 [34], which is functionally comparable to international standards such as SAE J661 [35] for evaluating the tribological properties of automotive friction materials. The friction and wear tests were performed within a temperature range of 100 °C to 350 °C. In this study, gray cast iron rotating disks with a hardness of HB 180–220 were employed as the counterface. During the testing process, the friction pair operated at a constant rotational speed of 480 rpm and a fixed pressure of 0.98 MPa. The detailed experimental procedures have been previously reported in our published works [18,36].

Prior to the commencement of the formal test, a running-in test is first conducted. The test specimen is subjected to initial grinding at temperatures below 100 °C until the contact area between the specimen and the friction disk reaches 95% or more. In the aging test, the friction specimen is tested at six consecutive rising temperatures of 100 °C, 150 °C, 200 °C, 250 °C, 300 °C and 350 °C. The friction and wear tester rotates for 5000 revolutions at each temperature. In the recovery test, the friction specimen is measured at five temperatures: 300 °C, 250 °C, 200 °C, 150 °C and 100 °C. Furthermore, the friction and wear tester rotates for 1500 revolutions at each corresponding temperature. At least five replicate tests are conducted under each temperature condition.

The following formulae are used to calculate the coefficient of friction and the wear rate.

(1) $$\mu =\frac{f}{P\;}$$

(2) $$W=\frac{S(h_{1}-h_{2})}{2\pi RNf}$$

In the equation, *μ* represents the coefficient of friction, *W* represents the wear rate, *f* represents the average friction force during the test, and *P* represents the normal force acting on the specimen, the working pressure. *R* is the distance between the center of the rotating disk and the center of the friction material specimen (150 mm); *A* is the total friction area of the specimen under test (625 mm^2^); *N* is the total number of revolutions of the rotating disk during the friction and wear test (*N* = 5000); *h_1_* is the average specimen thickness before the friction and wear test (mm); and *h_2_* is the average specimen thickness after the friction and wear test (mm).

As friction is a dynamic process, the thermal fade resistance and recovery performance of friction materials are key indicators for assessing their effectiveness and reliability during braking operations [36]. The fade rate and recovery rate of friction material specimens are calculated using the equation.

(3) $$R_{fade}=\frac{\mu_{F100}-\mu_{F350}}{\mu_{F100}}\times 100\%$$

(4) $$R_{recovery}=\frac{\mu_{R100}}{\mu_{F100}}\times 100\%$$

In the equation, *R_fade_* represents the fade rate of the friction specimen (%), and *R_recovery_* represents the recovery rate of the friction specimen (%). *μ_F*100*_* and *μ_F*350*_* denote the coefficients of friction at 100 °C and 350 °C, respective, during the fade test, whilst *μ_R*100*_* denotes the coefficient of friction at 100 °C during the recovery test.

### 2.4. Wear Surface Morphology Characterization

The worn surfaces and swabs of the specimens were characterized using a scanning electron microscope (SEM, TESCAN VEGA3, Brno, Czech Republic). As the friction material specimens have low electrical conductivity, all specimens were gold-sputtered.

## 3. Results and Discussion

### 3.1. Physical and Mechanical Properties’ Analysis

**Figure 3:**
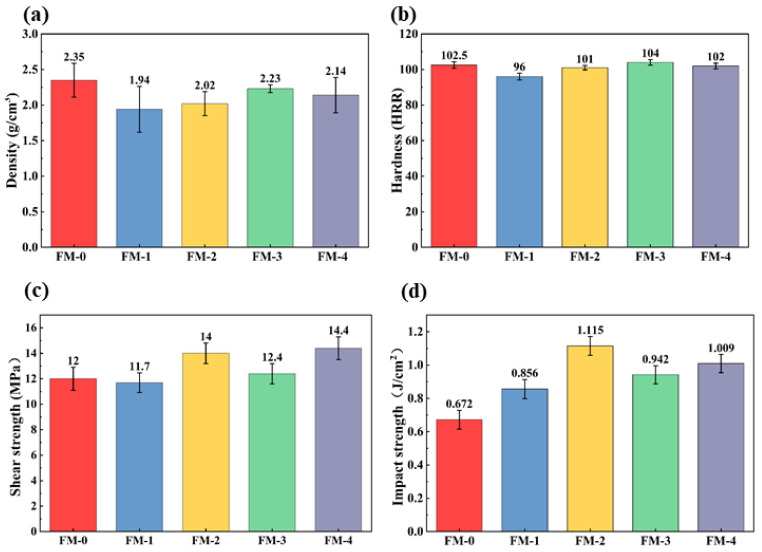
Physical and mechanical properties of braking friction materials: (**a**) Density, (**b**) Hardness, (**c**) Shear Strength, and (**d**) Impact Strength.

Figure 3 illustrates the experimental results for the density, hardness, shear strength, and impact strength of the FM-0, FM-1, FM-2, FM-3, and FM-4 brake friction materials. Regarding the physical properties of the specimens, as depicted in Figure 3a, the density of all agricultural waste-based composites falls within the range of 1.94–2.23 g/cm^3^, which is significantly lower than that of FM-0. This observation indicates that the replacement of high-density copper fibers with low-density biomass fibers can achieve a 14.9% reduction in the weight of the friction material.

The Rockwell hardness of the composite materials is shown in Figure 3b. The order of hardness is FM-1 < FM-2 < FM-4 < FM-0 < FM-3. Among these, FM-3 has the highest hardness, reaching 104 HRR. This is primarily attributed to the naturally occurring high-hardness silicon dioxide (SiO_2_) particles in rice husks. These particles act as hard support points distributed within the polymer matrix. In contrast, FM-1 and FM-2 exhibit lower hardness. This may be related to their higher internal porosity and the lower intrinsic modulus of the fibers. This characteristic may confer better adaptability at the friction interface.

The mechanical properties of the brake friction material specimen are shown in Figure 3c,d. The shear strength of the specimens is shown in Figure 3c. The shear strengths of FM-4 and FM-2 were 14.4 MPa and 14.0 MPa, respectively, which were superior to those of the other specimens. This is because sugarcane bagasse and wheat straw possess fibers with a relatively high aspect ratio. Physical and mechanical interlocking has enhanced the interfacial adhesion [37]. This structure facilitates the effective transfer of load from the matrix to the fibers, thereby significantly improving the material’s resistance to shear failure [38].

The impact properties of the specimens are shown in Figure 3d. The impact resistance of the specimens varies significantly depending on the type of waste. Among these, FM-2 and FM-4 exhibited the highest impact strength, representing a 39.7% increase compared to FM-0. Although the rice husk group exhibited high hardness, its impact strength was relatively low due to the limited ability of the granular components to hinder crack propagation, demonstrating certain brittle characteristics. The physical and mechanical properties of the specimens in this group all comply with national standards.

### 3.2. Coefficient of Friction Analysis

**Figure 4:**
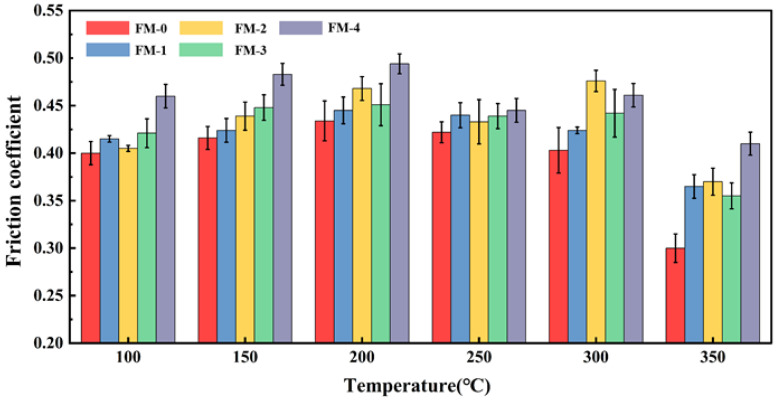
Friction coefficient of brake friction materials during fade tests.

**Figure 5:**
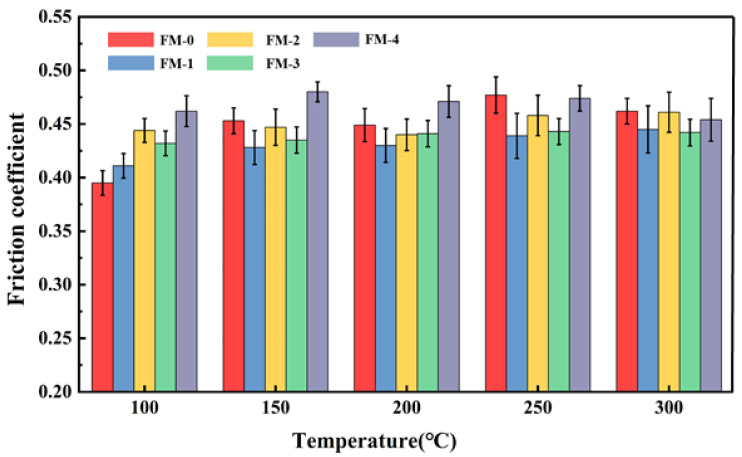
Friction coefficient of brake friction materials during recovery test.

The stability of the coefficient of friction (COF) serves as a critical indicator for assessing the thermal safety of friction materials; its stability directly dictates the reliability of the braking system under complex operating conditions. In this study, the effects of various agricultural waste fillers on the friction properties of composite materials during heating and cooling cycles were systematically investigated at a constant rotational speed of 480 rpm and an atmospheric pressure of 0.98 MPa. Figure 4 and Figure 5, respectively, illustrate the variation in COF for friction material specimens under fade and recovery tests.

During the heating phase of the test, as depicted in Figure 4, the COF trends for each specimen exhibited a high degree of consistency. Notably, the COF for all specimens incorporated with agricultural waste was significantly higher than that of the reference specimen FM-0. This observation indicates that the synergistic system comprising agricultural waste and carbon fibers exerts a substantial enhancing effect on the friction coefficient of the material.

Within the temperature range of 100 °C to 200 °C, the coefficient of friction (COF) for all friction specimens exhibited an upward trend. This phenomenon is primarily attributed to the running-in effect on the friction pair surfaces during the initial stage, as well as the glass transition of the phenolic resin matrix within this temperature interval, which increases the effective contact area. As the temperature rose further to 250 °C, the COF began to decline. Upon reaching 350 °C, the COF of the FM-0 specimen decreased sharply to 0.30, manifesting severe thermal instability. In contrast, the systems incorporating agricultural waste combined with carbon fibers maintained a high friction level under these elevated temperature conditions, demonstrating remarkable thermal stability.

It is noteworthy that the friction coefficient of each specimen exhibits distinct trends within the temperature range of 250 °C to 300 °C. The FM-0 specimen shows a declining friction coefficient due to the thermal decomposition of the resin matrix, which triggers interfacial thermal debonding and induces thermal fade. In contrast, specimens containing agricultural waste demonstrate enhanced frictional stability. For the FM-1 specimen (the corncob group), the high hemicellulose content of the corncob leads to a rapid pyrolysis rate within this temperature range, resulting in a slight decrease in the friction coefficient [39]. However, the carbon fibers promptly provide high-temperature skeletal support to effectively inhibit the fading process. The friction coefficients of the remaining specimens show an upward trend, primarily attributed to the synergistic effect between biomass carbonization products and carbon fibers. This effect promotes the formation of a secondary friction film, which effectively suppresses the thermal fade phenomenon. These comparative results indicate that the composite system of agricultural waste and carbon fiber possesses superior thermal fade resistance within the 250 °C to 300 °C range.

As shown in Figure 5, the friction coefficients of the specimens during the cooling and recovery process remained stable, with no significant fluctuations observed in any specimen except for the FM-0 specimen. Furthermore, at 100 °C, the friction coefficients of all specimens were slightly higher than their initial friction levels. This indicates that the composite materials comprising agricultural waste and carbon fibers exhibit excellent recovery properties and friction stability. It should be emphasized that the coefficient of friction for all copper-free friction materials developed in this study remains within the range of 0.35–0.48 across the entire temperature range, fully satisfying the requirements of the valid range specified in the Chinese national standard GB 5763-2018 [34].

### 3.3. Fade and Recovery Properties’ Analysis

**Figure 6:**
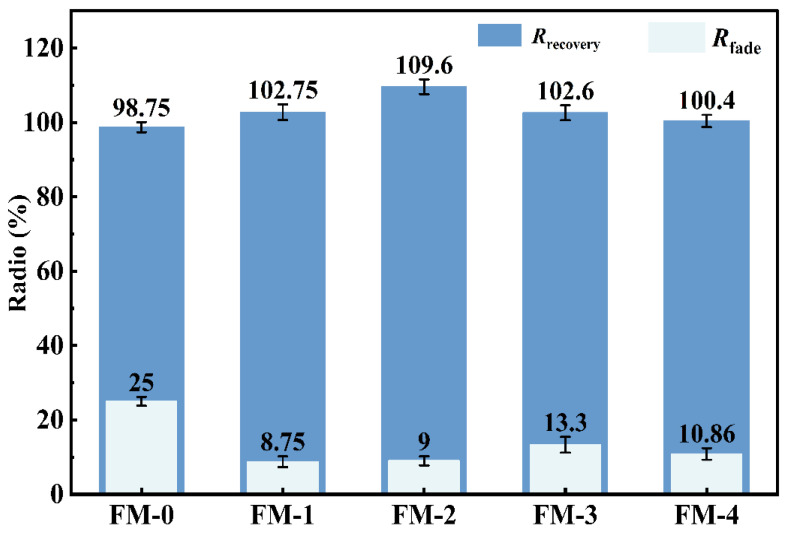
Fade and recovery performance of braking friction materials.

Fade performance and recovery performance are key indicators for assessing changes in friction force in friction materials at high temperatures. Brake friction materials must exhibit a relatively low fade rate and a high recovery rate [13]. Friction materials with different formulations demonstrate significant differences in terms of fade performance and recovery performance. Figure 6 shows the fade and recovery characteristics of FM-0, FM-1, FM-2, FM-3 and FM-4.

The fading rates of the five specimens, in descending order, are: FM-1 < FM-2 < FM-4 < FM-3 < FM-0. The fading rate of FM-0 is as high as 25%, which is higher than that of the other specimens. This indicates that traditional metal fillers are at significant risk of friction force degradation under high-temperature conditions. In contrast, the degradation rates of FM-1 and FM-2 were as low as 8.75% and 9.00%, respectively, far superior to the other components. This indicates that they possess better thermal degradation resistance and friction stability [39,40]. In terms of recovery performance, the recovery rates of the five specimens were, in descending order: FM-2 > FM-1 > FM-3 > FM-4 > FM-0. FM-2 exhibited the highest recovery rate at 109.60%. This indicates optimal recovery performance. Furthermore, the recovery rates of FM-1, FM-3 and FM-4 were all above 100%.

In summary, both the FM-1 and FM-2 specimens demonstrated excellent friction stability during high-temperature fade and recovery testing, with a fade rate of only around 9% at 350 °C and a recovery rate exceeding 100%. These results validate the feasibility of this copper-free green formulation for use in high-performance braking applications.

### 3.4. Wear Rate Analysis

**Figure 7:**
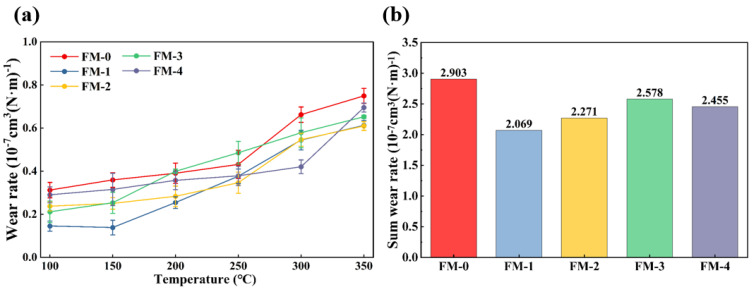
Wear properties of braking friction material specimens: (**a**) Wear rate and (**b**) Sum wear rate.

The wear resistance of brake friction materials significantly dictates their service life and overall performance, being fundamentally governed by the chemical composition and physical structure of the constituent fillers. This study characterizes and compares the wear resistance of brake friction materials incorporated with five distinct types of agricultural waste fillers. The experimental results, as presented in Figure 7, illustrate the instantaneous wear rate and the total wear rate. The materials containing various agricultural waste fillers exhibit divergent wear behaviors and evolution patterns across the specified test temperature range.

Figure 7a displays the instantaneous wear rates of the five specimens during the wear tests. The wear rates of all specimens increased in correlation with rising temperatures, indicating that the test temperature exerts a substantial influence on the wear performance of the specimens. This observation aligns with existing literature [13]. In general, the instantaneous wear rates of the FM-1 and FM-2 specimens remained consistently lower than those of the FM-0 specimen. Within the low-temperature regime from 100 to 250 °C, the wear rates of the specimens exhibited only marginal fluctuations. Notably, the wear rate of FM-1 remained at an exceptionally low level, which was significantly lower than that of the other specimens. This phenomenon is attributed to the inherent porous structure of the corn cob, which effectively captures hard particles at the friction interface, thereby mitigating abrasive wear. When the temperature exceeded 250 °C, the wear rates began to fluctuate due to the thermal decomposition and carbonization of the resin matrix and agricultural wastes [41,42,43]. As the degradation of the matrix accelerated, a pronounced modulus mismatch occurred between the metallic fibers and the resin matrix in the FM-0 specimen. This disparity induced interfacial delamination at elevated temperatures, leading to intensified material spalling and a subsequent substantial increase in the wear rate. The wear rates of the remaining specimens were lower than those of FM-0, owing to the high-temperature stability of the carbon fibers. Furthermore, although the rice husks in the FM-3 specimen possess high hardness, their intrinsic brittleness leads to microscopic fracturing under high shear stress. The resulting detached hard SiO_2_ particles act as third-body abrasives, which exacerbates abrasive wear on the friction surface [44]. The wear rate trend of the FM-4 specimen is situated between these two extremes.

The total wear rate is shown in Figure 7b, with the five specimens ranked in the following order: FM-1 < FM-2 < FM-4 < FM-3 < FM-0. The total wear rates of the remaining four groups of specimens were all lower than that of FM-0. This indicates that the introduction of a synergistic system combining agricultural waste and carbon fibers can effectively reduce the wear of friction materials. Compared with the FM-0 group, the wear rate of FM-2 decreased from 2.903 × 10^−7^ cm^3^/(N·m) to 2.069 × 10^−7^ cm^3^/(N·m), a reduction of 28.7%. This may be attributed to the excellent adhesion between the biomass fibers, carbon fibers and phenolic resin, which effectively reduces the tendency for components to be pulled out of the matrix.

In summary, composite materials combining agricultural waste with carbon fiber can effectively reduce the wear rate of test specimens, with corn cobs and wheat straw demonstrating the best performance in terms of wear resistance.

### 3.5. Friction Wear Mechanism Analysis

**Figure 8:**
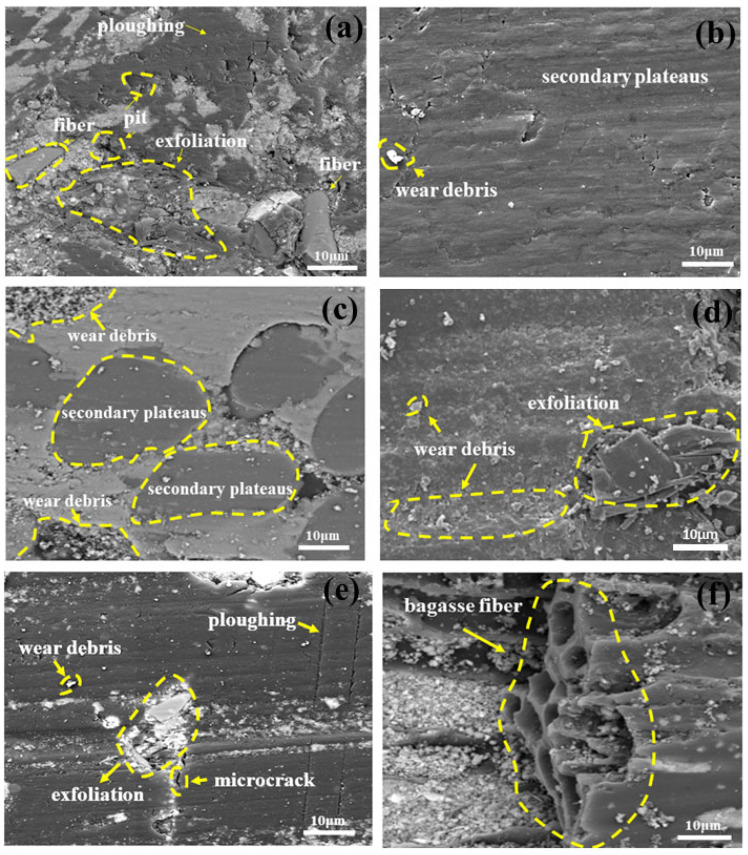
SEM micrographs of worn surfaces for braking friction materials: (**a**) FM-0, (**b**) FM-1, (**c**) FM-2, (**d**) FM-3, and (**e**,**f**) FM-4.

The evolution of the surface morphology of friction materials provides direct evidence for elucidating the synergistic mechanisms of their internal components and the underlying wear mechanisms. To investigate the influence of agricultural waste combined with carbon fibers on these wear mechanisms, Scanning Electron Microscopy (SEM) was employed to examine the surface morphology of the worn specimens. Figure 8 presents the SEM images of the wear surfaces of specimens FM-0, FM-1, FM-2, FM-3, and FM-4 following high-temperature testing at 350 °C. The observations reveal that the wear surface of the control group, FM-0, is the most rugged and lacks continuity, exhibiting a substantial number of exfoliation pits and plowing grooves. In contrast, the wear surfaces of the remaining specimens are relatively smooth.

The friction surface of the FM-0 specimen is illustrated in Figure 8a, exhibiting typical characteristics of brittle fracture and adhesive tearing. Observations reveal that the surface of the specimen is covered with plowing marks, exposed fibers, distinct fiber pull-out pits, and wear debris. This morphology is attributed to the significant modulus mismatch between the copper fibers and the resin matrix under high-temperature conditions, which results in a weakened interfacial bond. Under the coupled action of thermal and shear stresses, severe delamination occurs at the interface. Once the matrix loses the structural support provided by the fibers, it undergoes macroscopic delamination. This phenomenon subsequently triggers severe abrasive wear. The friction surface of the FM-3 specimen is presented in Figure 8d. It displays a surface characterized by numerous pits, delamination, and a substantial accumulation of abrasive debris, which are indicative of combined abrasive and adhesive wear. Although the FM-3 specimen possesses high hardness, the naturally occurring SiO_2_ particles within the filler are inherently brittle. Under cyclic loading, these hard phases are prone to stress concentration and are highly susceptible to fracturing. The detachment of hard debris not only compromises the integrity of the friction film but also induces severe fatigue wear. Particularly under elevated temperature conditions, as hard particles continue to shed and mix with voluminous friction debris, they repeatedly abrade the interface, thereby exacerbating surface spalling and causing a significant rise in the wear rate of the specimens. This unstable interfacial condition serves as the primary cause of fluctuations in the coefficient of friction. Consequently, the total wear rates for FM-0 and FM-3 remain relatively high, which is consistent with the previously discussed analysis of total wear rates.

Compared with the FM-0 specimen, FM-1 and FM-2 exhibited the smoothest and densest friction surfaces. A schematic diagram of the worn surface of FM-1 is shown in Figure 8b. Its worn surface is relatively smooth; apart from a small amount of fine debris, it is covered by a substantial friction film, i.e., a secondary contact plate, and no obvious interfacial delamination was observed [45]. Of all the specimens, FM-1 exhibited the least wear, which is consistent with the lowest wear rate recorded for this material. The formation of these secondary contact platforms is primarily attributable to the interfacial compatibility and adhesion between the maize cob structure and the resin. Under high-temperature conditions, the carbon fibers are firmly anchored within the matrix due to their high modulus and thermal stability [46]. They bear the majority of the normal loads and shear stresses, thereby preventing the matrix from collapsing. Following high-temperature carbonization, the maize cob bonds with the carbon fibers and is compacted, thereby forming a large number of secondary contact platforms. The formation of a secondary contact platform not only ensures the stability of the specimen’s friction properties but also reduces shear damage to the material surface caused by the friction pair, thereby enhancing the material’s wear resistance [47,48]. It effectively prevents the mating disk from directly cutting into the soft substrate, enabling the FM-1 to maintain minimal volume loss at high temperatures.

The worn surface of the FM-2 specimen is shown in Figure 8c, which reveals the presence of a secondary contact platform alongside a significant amount of wear debris and a small amount of exfoliation. This may be attributed to the fact that wheat straw fibers possess higher shear strength and hardness compared to corn cobs. Consequently, following repeated friction, the exfoliated fragments exert a cutting action on the worn surface. Consequently, the wear rate of the FM-2 specimen is slightly higher than that of the FM-1 specimen. This morphological characteristic is consistent with the analysis of physical and mechanical properties and wear rates discussed earlier, further revealing the microscopic differences in the influence of different agricultural waste components on the friction and wear behavior of the material.

Figure 8e shows the surface morphology of the FM-4 specimen after wear, with a surface covered in numerous wear debris, cracks and exfoliation phenomena, indicative of abrasive and fatigue wear. The primary cause of this phenomenon lies in the intrinsic properties of the bagasse fibers [49]. Due to their high aspect ratio, the bagasse fibers effectively enhance the matrix’s shear strength. This is consistent with the experimental results mentioned earlier, which showed that FM-4 exhibited the highest shear strength.

However, at high temperatures of 350 °C, thermal stress concentration at the interface leads to a reduction in interfacial strength. During frequent and repeated friction, some of the sugarcane bagasse fibers are pulled out of the matrix, causing the local reinforcement effect to fail. The SEM image in Figure 8f also supports this view. Clear evidence of fibers being torn and separated from the matrix was observed on the surface. Furthermore, the debris and sharp fracture surfaces formed upon fiber breakage further exacerbated abrasive wear and induced surface cracking. Nevertheless, compared to the surface of the FM-0 specimen, the surface of the FM-4 specimen retained a localized friction layer, demonstrating a moderate level of wear resistance.

In summary, the synergistic effect between agricultural waste and carbon fibers significantly improves the surface quality of friction materials and induces the formation of a primary contact platform. Experimental results indicate that this synergistic mechanism maintains the integrity of the friction surface under high-temperature conditions through effective interfacial restructuring, thereby substantially reducing wear.

## 4. Conclusions

The integration of agricultural waste with carbon fibers as a substitute for copper was evaluated across various formulations, leading to the following conclusions:

1. This study confirms the feasibility of utilizing agricultural wastes—specifically corncob, wheat straw, rice husk, and sugarcane bagasse—in synergy with carbon fibers to develop environmentally friendly, copper-free friction materials. Experimental results demonstrate that the structural support provided by carbon fibers compensates for the inherent strength limitations of agricultural waste at elevated temperatures. This synergistic system maintains stable tribological performance even under extreme conditions of 350 °C. The friction coefficient remains consistent within the range of 0.35 to 0.48, exhibiting a distinct competitive advantage in thermal fade resistance.
2. In comparison with the control group, the wear rates of the composite specimens decreased by 28.73%, 21.77%, 15.43%, and 11.20%, respectively. Among the biomass filler systems investigated, the composite modified with corncob achieved the most significant reduction in wear rate, reaching 28.73%. This indicates that the combination of corncob filler and high-strength carbon fibers exerts a positive synergistic effect, which substantially inhibits material degradation and reduces the rate of wear. Consequently, it is confirmed that the synergy between agricultural waste and carbon fibers effectively enhances the wear resistance of the friction materials.
3. This synergistic modification mechanism significantly improves the thermal fade resistance of the composites. Compared to the control group, the friction recovery rate of the specimens increased by an average of 5.15%, while the thermal fade rate decreased by an average of 48.09%, demonstrating superior high-temperature thermal stability. These findings indicate that the synergy between agricultural waste fillers and carbon fibers enhances resistance to thermal fade, serving as a viable formulation system for the development of high-performance copper-free friction materials.
4. This study proposes a novel design strategy for copper-free friction materials centered on the high-value utilization of low-cost resources. This approach provides a critical engineering pathway for the future development of braking materials that integrate economic efficiency, environmental sustainability, and high mechanical performance.

## Figures and Tables

**Table 1:** Composition of braking friction material components.

Component	Materials	Specifications	Manufacturer
Substrate	Phenolic resin	200 meshes	Shijiazhuang Mayue Building Materials Co., Ltd., Shijiazhuang, China
Reinforced fiber	Compound mineral fibers	3 mm	Shijiazhuang Mayue Building Materials Co., Ltd., Shijiazhuang, China
	Carbon fiber	3 mm	Changzhou Chuangying New Material Technology Co., Ltd., Changzhou, China
	Agricultural waste powder	100 meshes	Changchun, China
	Copper fiber	3 mm	Changchun Tebike Shili Auto Parts Co., Ltd., Changchun, China
Performance regulator	Aluminum oxide	600 meshes	Henan Borun CastingMaterial Co., Ltd., Gongyi, China
	Flake Graphite	325 meshes	Shandong Antu Brake Materials Co., Ltd., Taian, China
	Petroleum coke	400 meshes	Shijiazhuang Mayue Building Materials Co., Ltd., Shijiazhuang, China
	Friction power	80 meshes	Shandong Antu Brake Materials Co., Ltd., Taian, China
	Nitrile rubber powder	200 meshes	Shandong Antu Brake Materials Co., Ltd., Taian, China
	Zirconium silicate	500 meshes	Changchun Tebike Shili Auto Parts Co., Ltd., Changchun, China
	Barium sulfate	1250 meshes	Shandong Yusuo Chemical Technology Co., Ltd., Linyi, China
	Calcium carbonate	1250 meshes	Shandong Yusuo Chemical Technology Co., Ltd., Linyi, China
	Vermiculite powder	100 meshes	Lingshou Xuyang Mining Co., Ltd., Shijiazhuang, China
	Zinc stearate	325 meshes	Changchun Tebike Shili Auto Parts Co., Ltd., Changchun, China

**Table 2:** Formulation of the friction materials.

Ingredients	FM-0	FM-1	FM-2	FM-3	FM-4
Phenolic resin (wt%)	14	14	14	14	14
Carbon fiber (wt%)	6	6	6	6	6
Compound mineral fiber (wt%)	10	10	10	10	10
Copper fiber (wt%)	6	0	0	0	0
Corn cob powder (wt%)	0	6	0	0	0
Wheat straw powder (wt%)	0	0	6	0	0
Rice husk powder (wt%)	0	0	0	6	0
Sugarcane bagasse powder (wt%)	0	0	0	0	6
Performance regulator (wt%)	64	64	64	64	64

## Data Availability

The original contributions presented in this study are included in the article. Further inquiries can be directed to the corresponding author.

## References

[1] Sathyamoorthy G., Raghunathan V., Rangappa S.M., Siengchin S., Singaravelu D.L. (2025). Exploring the tribological impact of micaceous additives in copper-free automobile brake friction composites. J. Vinyl Addit. Technol..

[2] Kumar M., Bijwe J. (2011). Non-asbestos organic (NAO) friction composites: Role of copper; its shape and amount. Wear.

[3] Parkin J.G.H., Dean L.S.N. (2025). Copper-enriched automotive brake wear particles perturb human alveolar cellular homeostasis. Part. Fibre Toxicol..

[4] Sandahl J.F., Baldwin D.H., Jenkins J.J., Scholz N.L. (2007). A sensory system at the interface between urban stormwater runoff and salmon survival. Environ. Sci. Technol..

[5] Straffelini G., Ciudin R., Ciotti A., Gialanella S. (2015). Present knowledge and perspectives on the role of copper in brake materials and related environmental issues: A critical assessment. Environ. Pollut..

[6] Tarasiuk W., Golak K., Tsybrii Y., Nosko O. (2020). Correlations between the wear of car brake friction materials and airborne wear particle emissions. Wear.

[7] Zheng K., Min Z., Zhang F., Ren Z., Lin Y. (2025). High Heat-fade Resistance, Metal-free Resin-based Brake Pads: A Step towards Replacing Copper by Using Andalusite. Chin. J. Mech. Eng..

[8] Joshi A.G.G., Bharath K.N., Basavarajappa S. (2023). Recent progress in the research on natural com-posite brake pads: A comprehensive review. Tribol.-Mater. Surf. Interfaces.

[9] Sugozu B., Erol E., Sugozu I. (2024). Tribological and thermal characteristics of copper-free brake friction composites. Mater. Test..

[10] Vijay R., Singaravelu D.L., Jayaganthan R. (2020). Development and characterization of stainless steel fiber-based copper-free brake liner formulation: A positive solution for steel fiber replacement. Friction.

[11] Pujar N.M., Mani Y., Mouleeswaran S. (2022). Experimental investigation on three-body abrasive wear behaviour of novel natural cellulosic pigeon pea stalk fibre reinforced epoxy biocomposites. Mater. Res. Express.

[12] Wei L., Choy Y.S., Cheung C.S., Jin D. (2020). Tribology performance, airborne particle emissions and brake squeal noise of copper-free friction materials. Wear.

[13] Ma Y.H., Liu Y.C., Ma S.S., Wang H.B., Gao Z.H., Sun J.J., Tong J., Guo L. (2014). Friction and Wear Properties of Dumbbell-Shaped Jute Fiber-Reinforced Friction Materials. J. Appl. Polym. Sci..

[14] Öztürk B., Arslan F., Öztürk S. (2013). Effects of Different Kinds of Fibers on Mechanical and Tribological Properties of Brake Friction Materials. Tribol. Trans..

[15] Karthikeyan M.K.V., Raghuvaran S., Girisha L., Kharche N.A., Venkatesh R., Prabagaran S., Soudagar M.E.M., Obaid S.A., Alharbi S.A. (2024). Influences of silicon carbide nanoparticles on graphite reinforced sisal (agave sisalana) fiber hybrid composite: Behaviour study. J. Mech. Sci. Technol..

[16] Antonyraj I.J., Vijay R., Sathyamoorthy G., Singaravelu D.L. (2023). Influence of graphite purity concentrations on the tribological performance of non-asbestos organic copper-free brake pads. Ind. Lubr. Tribol..

[17] Raghunathan V., Sathyamoorthy G., Ayyappan V., Singaravelu D.L., Rangappa S.M., Siengchin S. (2024). Effective utilization of surface-processed/untreated Cardiospermum halicababum agro-waste fiber for automobile brake pads and its tribological performance. Tribol. Int..

[18] Ma Y.H., Wu S.Y., Zhuang J., Tong J., Xiao Y., Qi H.Y. (2018). The Evaluation of Physio-Mechanical and Tribological Characterization of Friction Composites Reinforced by Waste Corn Stalk. Materials.

[19] Bakry M., Mousa M.O., Ali W.Y. (2013). Friction and wear of friction composites reinforced by natural fibres. Mater. Und Werkst..

[20] Carlevaris D., Leonardi M., Straffelini G., Gialanella S. (2023). Design of a friction material for brake pads based on rice husk and its derivatives. Wear.

[21] Amirjan M. (2019). Microstructure, wear and friction behavior of nanocomposite materials with natural ingredients. Tribol. Int..

[22] Adeyemi O.I., Kirwan K., Tuersley I., Coles S.R. (2024). Comparative assessment of the performance of friction materials based on different agricultural wastes. Tribol. Int..

[23] Kumar N., Mehta V., Kumar S., Grewal J.S., Ali S. (2022). Bamboo natural fiber and PAN fiber used as a reinforced brake friction material: Developed asbestos-free brake pads. Polym. Compos..

[24] Ossia C.V., Big-Alabo A. (2021). Development and characterization of green automotive brake pads from waste shells of giant African snail (*Achatina achatina* L.). Int. J. Adv. Des. Manuf. Technol..

[25] Oladele I.O., Ayanleye O.T., Adediran A.A., Makinde-Isola B.A., Taiwo A.S., Akinlabi E.T. (2020). Characterization of Wear and Physical Properties of Pawpaw-Glass Fiber Hybrid Reinforced Epoxy Composites for Structural Application. Fibers.

[26] Chan D., Stachowiak G.W. (2004). Review of automotive brake friction materials. Proc. Inst. Mech. Eng. D.

[27] Rani M., Choudhary P., Krishnan V., Zafar S. (2021). A review on recycling and reuse methods for carbon fiber/glass fiber composites waste from wind turbine blades. Compos. Part B Eng..

[28] Aranganathan N., Bijwe J., Khatri D.S. (2016). Role of combination of hexagonal boron nitride and graphite in NAO friction material. Proc. Inst. Mech. Eng. Part J J. Eng. Tribol..

[29] Wang Z.Q., Gao D.R. (2014). Friction and wear properties of stainless steel sliding against polyetheretherketone and carbon-fiber-reinforced polyetheretherketone under natural seawater lubrication. Mater. Des..

[30] Xian G., Walter R., Haupert F. (2006). Friction and wear of epoxy/TiO_2_ nanocomposites: Influence of additional short carbon fibers, Aramid and PTFE particles. Compos. Sci. Technol..

[31] Gao H.M. (2007). Mineral Composite Friction Material.

[32] (2017). Test Method of Impact Strength for Friction Material.

[33] (2023). Road Vehicles—Brake linings—Shear Test Method for Disc Brake Pad and Drum Brake Shoe Assemblies.

[34] (2018). Brake Linings for Automobiles.

[35] (2021). Brake Lining Quality Test Procedure.

[36] Ma Y.H., Wu S.Y., Tong J., Zhao X.L., Zhuang J., Liu Y.C., Qi H.Y. (2018). Tribological and mechanical behaviours of rattan-fibre-reinforced friction materials under dry sliding conditions. Mater. Res. Express.

[37] Ji Z.J., Luo W.Y., Zhou K.K., Hou S., Zhang Q.F., Li J.Y., Jin H.Y. (2018). Effects of the shapes and dimensions of mullite whisker on the friction and wear behaviors of resin-based friction materials. Wear.

[38] Yavas D. (2025). Enhancing Interlaminar Shear Strength in Additively Manufactured Carbon Fiber-Reinforced Thermoplastic Composites Through Microstructural Design. Exp. Mech..

[39] Singh S., Sawarkar A.N. (2021). Pyrolysis of corn cob: Physico-chemical characterization, thermal decomposition behavior and kinetic analysis. Chem. Product Process Model..

[40] Kiliç H. (2024). Performance assessment of agricultural waste based eco-friendly brake friction composites. Polym. Compos..

[41] Bijwe J., Nidhi, Majumdar N., Satapathy B.K. (2005). Influence of modified phenolic resins on the fade and recovery behavior of friction materials. Wear.

[42] Huang L.R., Huang M.J., Du J.H., Liu Z.G., Li W., Zhu J.B. (2024). Study on the Friction and Wear Properties of Multiple Rare-Earth-Oxide-Reinforced Resin-Based Friction Materials. Materials.

[43] Li X.Q., Jia X.H., Yang J., Li Y., Wang S.Z., Song H.J. (2022). Interfacial modification and tribological properties of ZnO nanosheet carbon fiber reinforced poly(hexahydrotriazine) composites. Tribol. Int..

[44] Gehlen G.S., Neis P.D., Barros L.Y., Poletto J.C., Ferreira N.F., Amico S.C. (2022). Tribological performance of eco-friendly friction materials with rice husk. Wear.

[45] Lee S., Jang H. (2018). Effect of plateau distribution on friction instability of brake friction materials. Wear.

[46] Chae Y.K., Lee S., Lee J., Lee J., Kim T.H., Choi J., Lee S. (2026). Decoupling crystallinity from thermal stability: Revisiting thermal resistance of PAN-based high modulus carbon fibers. Carbon Lett..

[47] Eriksson M., Jacobson S. (2000). Tribological surfaces of organic brake pads. Tribol. Int..

[48] Barros L.Y., Poletto J.C., Buneder D., Flores R., Gehlen G., Neis P.D., Ferreira N.F., Matozo L.T. (2021). An experimental study of the transition in the wear regime of brake friction materials. Polym. Compos..

[49] Demisie L.F., Getnet E., Taddese G.A., Ayalew Y.G., Ejigu A.A. (2025). Effect of the weight fraction of bagasse and aloe vera ash on the friction and sliding wear behavior of an Al6061 reinforced silicon carbide hybrid composite. Discov. Mater..

